# Reuse of terminological resources for efficient ontological engineering in Life Sciences

**DOI:** 10.1186/1471-2105-10-S10-S4

**Published:** 2009-10-01

**Authors:** Antonio Jimeno-Yepes, Ernesto Jiménez-Ruiz, Rafael Berlanga-Llavori, Dietrich Rebholz-Schuhmann

**Affiliations:** 1 European Bioinformatics Institute, Wellcome Trust Genome Campus, Hinxton, Cambridge, CB10 1SD UK; 2 Departamento de Lenguages y Sistemas Informáticos, Universitat Jaume I, Castellón de la Plana, 12071 Spain

**Keywords:** Juvenile Idiopathic Arthritis, Domain Ontology, Ontology Engineering, Open Biomedical Ontology, Shared Concept

## Abstract

This paper is intended to explore how to use terminological resources for ontology engineering. Nowadays there are several biomedical ontologies describing overlapping domains, but there is not a clear correspondence between the concepts that are supposed to be *equivalent* or just *similar*. These resources are quite precious but their integration and further development are expensive. Terminologies may support the ontological development in several stages of the lifecycle of the ontology; e.g. ontology integration. In this paper we investigate the use of terminological resources during the ontology lifecycle. We claim that the proper creation and use of a shared thesaurus is a cornerstone for the successful application of the Semantic Web technology within life sciences. Moreover, we have applied our approach to a real scenario, the Health-e-Child (HeC) project, and we have evaluated the impact of filtering and re-organizing several resources. As a result, we have created a reference thesaurus for this project, named **HeCTh**.

## Introduction

Large domain ontologies are emerging from collaborative efforts in the Life Sciences, being its main aim to achieve the interoperability among the different research resources by assuming a common conceptualization. These resources mainly consist of both domain ontologies and terminological resources (e.g. thesauri), which allow researchers to process, store and share the ever increasing knowledge derived from their experiments. So far, these two kinds of resources have usually lived apart, being its later integration a very hard task. However, some exceptions exist where the thesaurus is integrated within the ontology; e.g. the Open Biomedical Ontologies (OBO) [1] and the Foundational Model of Anatomy (FMA) [2] with the Terminologia Anatomica (TA) [3].

Unlike OBO ontologies, we propose a loose coupling between the domain ontologies and the reference thesaurus that is similar to the idea proposed in FMA and the Terminologia Anatomica. Along this paper we show that the use and maintenance of such a shared thesaurus will enable both a better integration of domain ontologies with existing terminological resources and the proper evolution of the thesaurus according to these ontologies. We claim that the use of a reference and shared thesaurus will ease some of the problems present during the development of ontologies and their interoperability.

In this paper we assume that ontologies and terminological resources have different purposes, and therefore they should not be treated with the same techniques nor simply merged into a common resource. A *lexicon* consists of a compendium of terms enriched with information on its usage [4], being concerned with the linguistic properties of words. We may encounter as well the term *terminology*, which is usually referred as a *specialized lexicon* [5]. A *thesaurus* could be considered similar to a lexicon but with different purposes. A thesaurus is not focused, in general, on linguistic properties, but on the organization of terms within a taxonomy (e.g. hypernymy). Finally, an *ontology* is an *explicit specification of a conceptualization* [6] providing a non ambiguous and formal representation of a domain. Domain ontologies have much more specific purposes than lexicons or thesaurus, as their intended consumers are computer applications rather than humans. Thus, ontologies do not need to be overloaded with variants of the terms they use. Instead, a link (for each concept) to a reference thesaurus should be provided.

**Figure 1:**
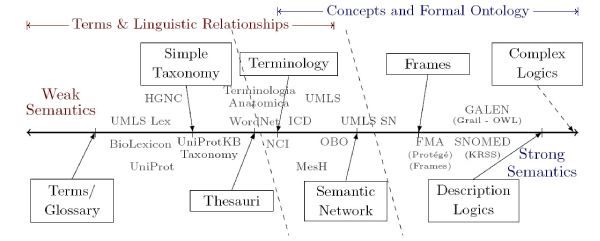
**Adapted Ontology Spectrum based on** [5, 59, 60].

In Figure 1 we have ordered the existing formalisms (denoted by boxes) according to their semantic expressiveness. Existing biomedical resources are placed to their closer formalism. Genuine lexical resources are placed closer to the left part of the diagram, like the Biolexicon [7], which contains terminology from several resources with some linguistic relevant information. We find as well the UMLS [8] Specialist lexicon that has been used within several NLP (natural language processing) and text mining applications. Closer to the limit between a lexicon and an ontology we find several resources that include links between lexical entries (e.g. UNIPROT). More complex resources lie in between the definition of ontology and lexicon like the NCI thesaurus, MeSH, ICD, the UMLS Metathesaurus (UMLS-Meta) and the OBO ontologies that account for more complex representations similar to semantic networks. Finally, at the end of the spectrum we find more formal ontologies such as FMA or Galen, which express stronger semantics. Unfortunately, formal ontologies usually lack links to lexical entries (i.e. reference to a thesaurus).

The selected examples and use cases presented in this paper come from the application domain of the EC FP6 Health-e-Child (HeC) project [9], which aims to develop an integrated health care platform for European paediatrics and decision support tools to access personalized health information. HeC project is mainly focused in paediatric heart diseases (e.g. Right Ventricular Overflow), inflammatory diseases (e.g. Juvenile Idiopathic Arthritis, JIA) and brain tumours. Within the objectives of HeC project several ontologies with different purposes are required to be created. These ontologies are intended to represent the involved knowledge by means of different levels of granularity: molecular (e.g. genomic and proteomic data), cellular (e.g. results of blood tests), tissue (e.g. synovial fluid tests), organ (e.g. affected joints, heart description), body (e.g. examinations, treatments), population (e.g. epidemiological studies). The purpose of this multilevel representation is to give a complete characterization of the different HeC diseases in order to provide a rich ontological layer to the HeC System. This semantic layer will be applied in *Data Integration* of heterogeneous sources, *Linkage* to external knowledge, *Query Enhancement* over the patient data, and in the *Decision Support Systems* for diagnosis, prognosis and follow-up [10, 11].

In this paper, we claim that the proper creation and use of a shared thesaurus is a cornerstone for the successful application of the Semantic Web technology. Moreover, we provide a review of current terminological resources, and how they should be reused for ontology engineering. As a proof of concept, we have created a reference thesaurus for HeC, named **HeCTh**.

## Discussion

**Figure 2:**
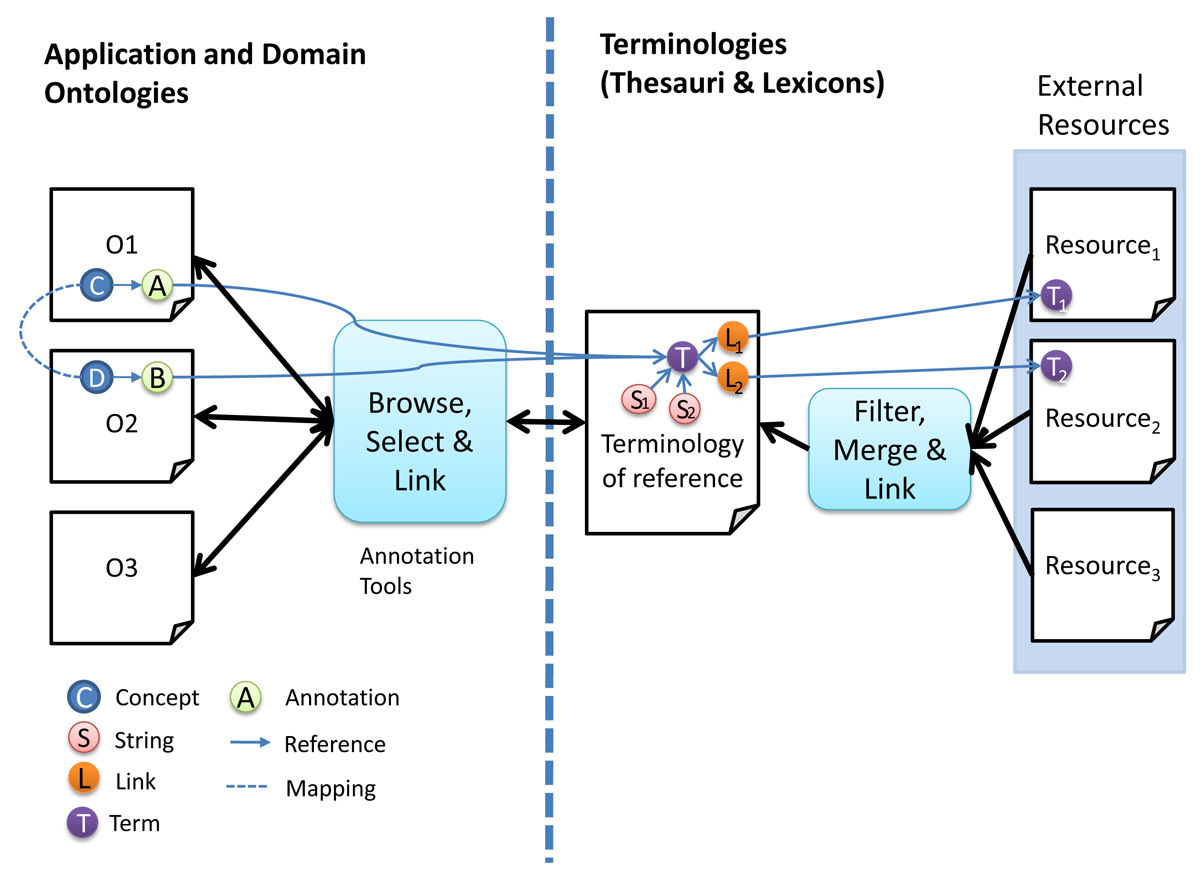
**Ontology thesaurus link**

Terminologies have been integrated in ontologies in different ways. In the most simple approach, the terminology is introduced directly as one of the properties of the concept. For example, the OBO ontologies have the terminology included as a part of the concept specification. Even though this approach is widely accepted by the community, we propose to keep the ontologies and terminological resources separated from each other since they have rather different purposes and lifecycles. Figure 2 shows an example of this setup. The concepts are linked to the thesaurus and, in some cases, we find that the same entry in the thesaurus is linked to several concepts. This may indicate that these two concepts can be potentially aligned. In addition, several entries in the thesaurus are linked to the same entry in the lexicon. This means that these terms are ambiguous. For instance, *retinoblastoma* can be either a disease or a gene and the ambiguity is easily detected.

### Thesauri-ontology linkage

In the proposed implementation, the thesaurus requires: a unique entry identifier, the link to the terms (synset) including the preferred term and a basic taxonomy that eases the handling of the thesaurus. The link of each entry to a *Semantic Category* (e.g. disease, gene, drug, organ, etc.) has been shown helpful for disambiguation purposes in many fields. Additionally, links to external resources, such as UMLS-Meta, can be also included in order to maintain a reference to the original thesaurus.

Furthermore, a specific setup in the terminology linked to the thesaurus may allow us to identify easily ambiguous terms. We would like to propose a further engineering improvement to the previous approach that is similar to the UMLS string representation. The terms linked to the thesaurus are stored in a table. The terms are linked to the entries in the thesaurus based on either synsets similar to WordNet or clusters as in the Biolexicon. Finally a property (e.g. entity annotation axioms in OWL) of the ontology links a concept to the entry in the thesaurus.

We aim at having an unique access point to the terminological resources. As mentioned in the introduction, this organization has several advantages that are emphasized in the following section, and which are mainly related to different stages of the ontology lifecycle (e.g. ontology integration). However, two issues should be properly addressed: (1) the difference between thesaurus integration and ontology integration, and (2) the maintenance of the linkage between ontologies and thesauri.

The efforts in thesauri integration and ontology integration provide ongoing and open discussions in both communities. Thesaurus alignment has a different purpose compared to ontology alignment since:

1. Thesauri are intended to contain terminological information, therefore the alignment of terms relies in most cases on term matching and do not perform a semantic analysis. For example, UMLS-Meta [12] is prominent project for integrating independent thesauri.
2. Ontologies represent the semantic layer of the domain, thus when integrating ontologies we should only care about semantic compatibility [13], and not about the normalization of the used terminology.

**Figure 3:**
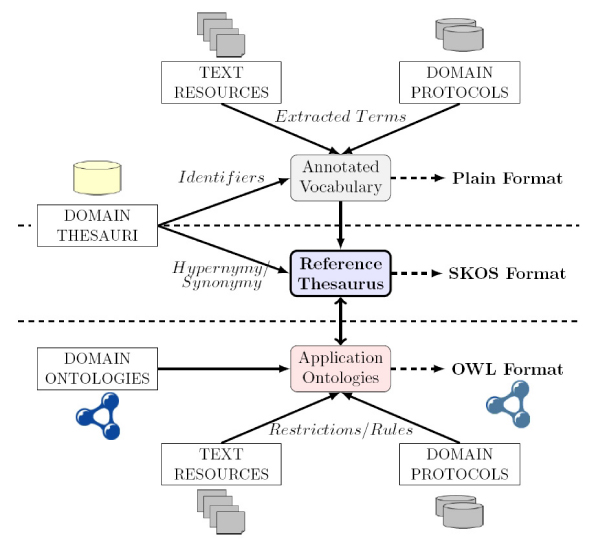
**From requirements to a reference Thesaurus and Ontologies**.

**Figure 4:**
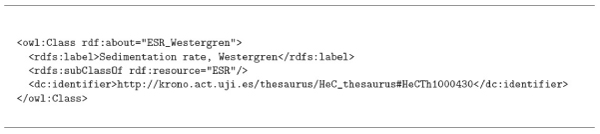
**Example of Ontology to Thesaurus mapping through an OWL Annotation**.

The ontology-thesaurus linkage (i.e. mapping) should be done using references or annotations provided by the ontology languages; in the following section and Figure 3 detailed information about the implementation of these links is presented. As an example, Figure 4 shows an OWL [14–16] annotation for the concept *ESR_Westergren*, which is linked to **HeCTh** term *HeCTh* 1000430 with preferred label *Sedimentation rate, Westergren*. Figure 2 shows an example where two concepts are annotated with the same thesaurus term, thus these two concepts can be potentially mapped if no semantic incompatibility is found.

Ontology editors should provide mechanisms to allow ontology engineers to browse thesauri and select the desired term in order to annotate their concepts. The UMLS Tab [17] for the ontology editor Protégé [18] was a good initiative trying to integrate UMLS-Meta within the ontology lifecycle. On the other hand, the OBO ontology editor [19] also allows cross references of defined concepts to synonyms coming from other resources.

### The role of a thesaurus in the ontology lifecycle

We have considered the METHONTOLOGY methodology [20] as the basis to illustrate how a shared thesaurus can help the development of an ontology and vice versa. METHONTOLOGY proposes several steps for the lifecycle of an ontology: Requirements Specification, Knowledge Acquisition, Conceptualization, Integration with top ontologies, Implementation, Evaluation and Evolution/Maintenance. Concretely, this section is intended to show problems that experts, knowledge engineers and ontology engineers find in the different stages of the lifecycle of the ontology development and how the use of a reference thesaurus could ease these problems.

**Figure 5:**
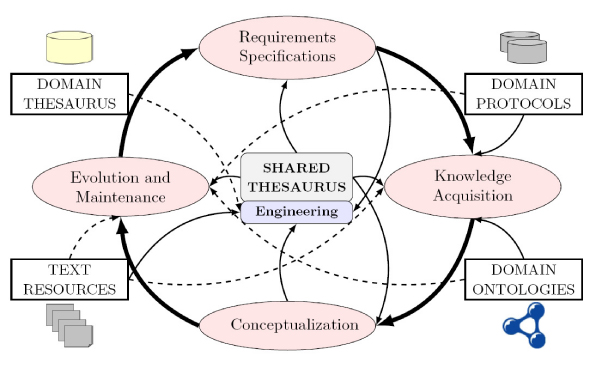
**The Thesaurus within the Ontology Life Cycle**. Solid arrows represent an essential role, whereas dashed arrows mean auxiliary role.

As Figure 5 shows, the shared thesaurus interacts with almost all the development phases (for our purposes we have focused on 4 phases, the other can be considered as a part of the *Conceptualization phase*).

Moreover, external resources like domain protocols, domain ontologies and research articles will also play an important role as sources of knowledge. In the following subsections we describe in detail the role of the thesaurus at each development phase. In addition, we present the issues identified in the HeC project, specifically within the Juvenile Idiopathic Arthritis (JIA) domain.

#### Requirements specification

JIA is a rare kind of Arthritis and there is not yet a consensus about its classification nor even its name [21]. So far, three classification schemes have been proposed, namely: ACR (American College of Rheumatology), which uses *Juvenile Rheumatoid Arthritis (JRA)* as preferred name and proposes three disease subtypes, EULAR (European League Against Rheumatism), which opts for *Juvenile Chronic Arthritis (JCA)* and proposes six disease subtypes, and finally ILAR (International League of Associations for Rheumatology) which prefers JIA and proposes eight subtypes.

In this stage, a classification criterion should be chosen and the initial set of terms for describing the disease and subtypes must be defined. Some concepts are hard to describe and the selection of a proper label for them is not always a straightforward task. The problem of label (i.e. term) selection, to better describe the ontology concepts without ambiguity, is well known by the community and already a topic of discussion in ontology development projects like *OpenGalen* [22]. Clearly, the use of a reference thesaurus would make easier the normalization and selection of terms for labeling the desired concepts.

On the other hand, the requirements specification for the ontology may imply the use of concept names not available as terms in the thesaurus (e.g. JIA subtypes names are not fully available and classified in UMLS-Meta) and therefore the thesaurus should evolve accordingly.

#### Knowledge acquisition

The knowledge acquisition in HeC is mainly based on a set of medical protocols (e.g. patient data forms) [23]. Each subtype of JIA is characterized by affecting different set and number of joints, the occurrence of some symptoms like fever or rash, the laboratory tests that are analyzed, the different treatments that are applied, etc. The development of the ontology from scratch would imply the conceptualization of the different joints of the body, the classification of the drugs for the treatments, the characterization of the different laboratory tests, etc. Nevertheless this knowledge is already well known by the community (unlike JIA) and it is assumed to be already defined in the available biomedical ontologies. As far as we know, the NCI thesaurus [24], the GALEN ontology [25] and the FMA ontology contain knowledge that is relevant to JIA.

The reuse of knowledge represented in ontologies (see [26] for a survey) could be interesting due to the following reasons: (a) developers save time through reusing existing ontologies rather than writing their own; (b) the used knowledge is commonly accepted by the community and used in similar applications; (c) developers are not always experts in all the areas covered by a concrete disease (i.e. drug classification). However, afore mentioned ontologies belong to independent projects and they do not use a common terminology (e.g. Galen contains little information about synonymy, some NCI concepts are linked to UMLS, and FMA uses *Terminologia Anatomica (TA)* [3]). Therefore, important drawbacks may arise when merging them. In this case, *Ontology Matching* [27] should be performed, that is, to discover the correspondences between entities of the different ontologies. This task is rather hard [28] since in most cases there is not a common terminology for the entity names. Some ontology matching techniques such as SAMBO [29] use external knowledge (i.e. UMLS) to discover non trivial mappings. Obviously, these techniques usually obtain the best precision and recall results, however they also required more computation time being non scalable for big ontologies (see the results of the Ontology Alignment Evaluation Initiative for the *Anatomy track* [30]). The linkage to a reference thesaurus and the use of term names as concept labels would relax considerably, besides providing better recall results, the required matching tasks between ontologies. Thus, ontology reuse techniques will only care about the *Semantic Compatibility* [13] (e.g. unsatisfiability, unintended consequences) of the ontologies to be integrated. At this point, ontology repair and reconciliation [31, 32] arise as new challenges, but they are outside the scope of this paper.

#### Conceptualization

The thesaurus should provide a consensual term for the concept label and the corresponding definition, nevertheless, not all terms or concepts can be described with words. As known, natural language could be rather ambiguous when describing complex and similar concepts. For this reason ontologies, and not thesauri, should provide logic based and non-ambiguous representations for these complex concepts. However, not all ontologies uses logic-based formalism to describe concepts, but a long concept name (e.g. GO ontology, Protein Ontology).

Regarding the classification, the thesaurus may provide an initial classification of the terms with some granularity level, which may be reused by the ontology. Nevertheless, the granularity of the ontology will be connected to the purposes of the application, in this sense, the same entry in a lexicon could have different interpretations/classifications within different ontologies. This characteristic is related to the *localized semantics* proposed in [33], in which the concept *context* is defined as local models representing a partial or concrete view of the domain. For our purposes the concepts and theory treated in [33] are rather complex but the general idea of the local use of a shared *concept* is important. For example, following the mentioned classification criteria, the concept *Chronic Childhood Arthritis* may have the interpretations given in axioms 1 to 3.123

Such interpretations belong to three different JIA ontologies used, probably, for different application purposes. If they are required to be integrated, the matching between labels would be straight forward if a shared thesaurus are used to annotate the concepts, that is, if *JIA*, *JCA* and *JRA* are linked to **HeCTh** term *HeCTh1000662* with preferred label *Juvenile Idiopathic Arthritis*. The semantic integration, as commented previously, will depend on the compatibility of the used axioms within the conceptualization and formalization of the merged JIA ontologies.

It is worth mentioning that the conceptualization requirements of an ontology may involve concepts with labels that are not present within the thesaurus. Perhaps, in this ontology lifecycle step the ontology will provide as knowledge to the thesaurus as the thesaurus to the ontology engineering process.

#### Evolution and maintenance

The evolution and maintenance (e.g. addition of new concepts, the deletion of obsolete ones, the re-structuring of the already defined concepts, the addition of new facts, etc.) of an ontology may be produced due to different reasons: requirements have changed, the domain has changed (e.g. new facts were discovered) or the point of view of the domain has changed (e.g. use of a different classification criterion). The evolutions will imply to come back to previous steps in order to acquire new knowledge and to integrate this knowledge within the ontology. Again, the thesaurus will play a key point providing the concepts necessities when possible or being updated with new ontology requirements in order to keep up-to-date for further ontology demands.

The evolution of the ontology may imply changes over the thesaurus like the addition of new entries, the deprecation of obsolete entries or the split of entries in several ones. Obviously the evolution of the thesaurus will also affect the referencing ontologies. For this reason, the thesaurus should release stable versions periodically if important changes were made. Moreover each entry of the thesaurus should also have metadata about the status of the entry, indicating if the entry is being reviewed (new entries), is obsolete (pointing to which entry or entries should be used instead), or just if the entry is up-to-date. Referencing ontologies should periodically check if the referenced version of thesaurus is the last one and if the used lexical entries suffered any change or become obsolete.

In biomedicine the change and extension of the domain evolves quickly. Publications represent an important source of *brand new* facts of domain knowledge. For example Medline [34] indexes more than 800,000 new journal papers per year containing the last research done in more than 700 topics. However several studies (e.g. [35]) have already shown that the link between the most relevant biomedical resources and the literature is not obvious. This is not only due to the complexity of the required matching algorithms but also due to the decouple of the ontology/thesaurus development effort and the literature. In an important number of cases current terminological resources do not provide useful synonyms to be detected within the text. In order to overcome these problems, thesauri should, at least, contain the synonyms with the variants used in texts.

### Limitations and drawbacks of current reference thesauri

Previous sections have introduced the proposed scenario where a thesaurus should be used as a reference for ontology engineering tasks. Currently there exists several thesauri with different purposes. We emphasize UMLS-Meta, the set of OBO Ontologies (OBO) and specialized resources with large terminologies like SwissProt knowledge base [36] and the DrugBank database [37].

The UMLS Metathesaurus (UMLS-Meta) represents the best effort for the creation of a multipurpose *reference thesaurus*. The UMLS-Meta contains concepts from more than 100 terminologies, classifications, and thesauri; e.g. FMA, MeSH, SNOMED CT or ICD. UMLS-Meta 2008AB includes almost 1.5 million terms and more than 3 million term names, it also provides hypernymy classification with more than 1 million relationships, moreover it also includes around 40 millions of other kinds of relationships.

Obviously, UMLS-Meta is a rich source of knowledge but with a high level of ambiguity and redundancy. In the literature, we can find some efforts [38, 39] to normalize the UMLS-Meta by filtering redundancy and solving a basic level of ambiguity [40]. However, UMLS-Meta still maintains several drawbacks due to its complexity.

OBO ontologies present an important community effort in the development of light-weight ontologies, being in the middle of what we expect from an ontology and a thesaurus. The underlying logic of the OBO ontologies is not too complex, being in most cases limited to taxonomies (e.g. *Disease Ontology*). Other OBO ontologies like *Gene Ontology* or *Protein Ontology* contains a large quantity of assertions but in most cases they refer to annotations (i.e. concept metadata).

SwissProt is a manually curated biological database of protein sequences which aims at providing reliable protein sequences associated with a high level of annotation (such as the description of the function of a protein, its domains structure, post-translational modifications, variants, etc.). On the other hand, DrugBank database combines detailed drug (i.e. chemical, pharmacological and pharmaceutical) data with comprehensive drug target (i.e. sequence, structure, pathway) information. Note that, unlike UMLS-Meta, both Swissprot and Drugbank represent specialized lexicons.

These resources represent very important efforts and somehow they are references within the bioinformatics community, however they could be refined and adapted in order to get a more useful *reference thesaurus for ontology engineers*. Next sections summarize the main drawbacks we have found for this purpose.

#### Ambiguity and lexical problems

Current domain thesauri contain a large number of complex term labels that surely will not have a correspondence neither in ontology concept labels nor texts. Next we present some representative cases that the intended reference thesaurus should avoid. Moreover, UMLS-Meta also contains ambiguity cases that will introduce noise in the selection of the proper term for an ontology concept or for a text entity.

##### Complex Ambiguity Cases

Some ambiguity cases are rather hard to solve. This is the case of the term *Prostate Cancer* which has associated two UMLS-Meta entries: *C* 0600139_*UMLS*_and *C* 0376358_*UMLS*_. Both concepts refer to the Neoplastic Processes, *Carcinoma of prostate* and *Malignant tumor of prostate*, respectively. These Neoplastic Processes have a close relationship, indeed the former is represented as a child of the later within the NCI and UMLS-Meta taxonomies.

##### Descriptive names

Some synonyms are closer to a text definition than to a term name. For example, UMLS-Meta *Therapeutic or Preventive* term *C* 0580168_*UMLS*_: *"Amputation of finger through distal interphalangeal joint"*. OBO ontologies also present similar definitions (e.g. Gene Ontology term *GO*: 0007180 *"transforming growth factor beta ligand binding to type II receptor"* (biological_process) or Protein Ontology term *PRO* _000000935 *"potassium/sodium hyperpolarization-activated cyclic nucleotide-gated channel 1 isoform 1 glycosylated form"*). Swissprot and DrugBank contain less cases but still some of the entries are hard to interpret (e.g. Drug *APRD* 00506_*DRUGBANK*_, *"calcium carbonate with vitamin d, magnesium, zinc, copper and manganese"*). Let us emphasize that not all concepts can be described with few words, indeed, such complex concepts should be described in formal ontologies by combining somehow smaller units of meaning of the thesaurus, e.g. term *C* 0580168_*UMLS*_can be formally described as *Amputation* ⊓∃*involve.Finger* ⊓∃*through.InterphalangealJoint*, where the semantics for each of its elements is defined in the formal ontology. Additionally, each of these concept constituents can be linked to entries of the thesaurus.

##### Parametrization in the label

The *Clinical Drug C* 1614077_*UMLS*_has the preferred name *"Etanercept 50 mg/mL subcutaneous solution"*. This term indicates not only the drug name but also the dosage for this pharmaceutical product. The thesaurus should contain only the generic name, and then the ontology should provide a formal representation of *C* 1614077_*UMLS*_as either a subclass of *"Etanercept"* (e.g. *Etanercept* _50 ⊑ *Etanercept* ⊓∃ *hasDosage*. "50 *mg/mL"*) or just as an instance.

#### Structural problems

UMLS-Meta is more complex than a thesaurus (closer to an ontology in some cases) and it does not only contain synonyms, hyponymy and hypernymy relationships (i.e. is-a or subsumption relationships in ontologies) but also other relationships like meronymy and holonymy (i.e. has-part, part-of). This makes UMLS-Meta really hard to process and explore since UMLS entries may have several parents and a huge number of ancestors. Moreover in some of the cases the taxonomy contains cycles. This is mainly due to the UMLS-Meta evolution strategy, which integrates several taxonomies and vocabularies where terms are not always classified following the same criterion. Within this evolution and integration process new terms are matched to existent ones or a new entry is created, in both cases the resulting classification is hard to determine. For example, *Chronic Childhood Arthritis* (*C* 0553662_*UMLS*_) has itself as a parent (i.e. broader term) and as child (i.e. narrower term) according to *SNOMED* and *ICD* – 10 classifications. On the other hand, as commented previously, OBO ontologies are between a thesaurus and an ontology. They also provide a fine granularity level of classification, being, in some cases, difficult to interpret and explore.

Although they are quite comprehensive vocabularies, Swissprot and DrugBank lack a rich classification scheme, being their organization limited to a set of families or categories.

Our proposed reference thesaurus should contain a clearer and not overloaded hierarchy with only *hypernymy* or *meronymy*. The granularity of the thesaurus hierarchy may vary from a top level classification (e.g. UMLS Semantic Network [41]) to fine granularity hierarchies like OBO classifications or the UMLS-Meta hypernymy hierarchy. Nevertheless, complex classification of the concepts should be delegated to the ontology conceptualization process.

## Methods

The proper creation of thesaurus entries requires the selection of the appropriate terms (i.e. preferred name, synonyms, hypernymys). These terms may be provided by a community effort, where several domain experts study the appropriate set of terms, and/or may be extracted from the scientific literature [42] using natural language processing (NLP) and text mining [43]. A reference thesaurus should provide *rich lexical and hierarchical information* about the domain, but without overloading the *quantity of information* to be processed.

In this section we present the steps that we followed to create a *light-weight thesaurus* for HeC (**HeCTh**), considering the indications of the previous section, so that it provides the lexical information required by the HeC domain and its application. We have reused and filtered UMLS-Meta, Swissprot and DrugBank in order to extract the necessary terms and relationships. Next, we distinguish three phases in the creation of this thesaurus (see Figure 3): vocabulary extraction, fragment extraction and thesaurus extension.

### Vocabulary extraction

As earlier commented, the information and knowledge acquisition in HeC is mainly based on a set of medical protocols [23]. These medical protocols provide different kind of data, from general patient information (e.g. gender, location, family history etc.) to examination data (e.g. physical examinations, images, laboratory tests etc...). Examinations are performed on patients during visits (e.g. baseline and several follow-ups) where each visit provides a context and purpose for the examinations. Moreover, every visit usually results in setting (or confirming) a diagnosis and/or suggesting some treatments. [23] proposed several techniques to automatically extract the main concepts from HeC protocols. For this purpose, we regard these medical protocols as a set of input controls (input fields in patient data forms), where each control has an associated text label (e.g. *Date of Diagnosis, Bone Erosion Evaluation (BEE)*). In [23] UMLS-Meta based annotations were used to assign an UMLS-Meta term, or a set of them (in case there is not an exact match), to each input form control.

In order to enrich the vocabulary given by medical protocols the literature was also mined in order to extract interesting terms related to HeC domain. Approaches presented in [39, 44, 45] analyzed different techniques to annotate textual resources with UMLS-Meta, Swissprot and DrugBank terms. [39] was mainly focused on the *term recognition evaluation* over disease names. Whereas [44] went further trying to analyze term co-occurrences within the *Juvenile Idiopathic Arthritis* domain in order to discover interesting relationships.

Together with the introduced automatic techniques, manual intervention was also necessary in order to polish obtained results giving the correspondent matching to the UMLS-Meta, Swissprot or DrugBank unique identifiers. Moreover, domain papers (e.g. [21, 46–48]) and web sites (e.g. Wikipedia [49]) have also been important to *manually* consider interesting terms and interesting criteria for patient classification. As a result a flat vocabulary [50] linked to the domain thesauri was obtained (see Table 1 for an excerpt).

**Table 1:** Excerpt from the HeC vocabulary format

Entry ID	Name	Thesauri Origin	External ID
*HeCTh* 1000014	*Joints*	*UMLS*	*C* 0022417
*HeCTh* 1000717	*c reactive protein*	*UMLS*:*SwissProt*	*C* 1413716:*P* 02741
*HeCTh* 1000788	*etanercept*	*UMLS*:*DrugBank*	*C* 0717758:*BIOD* 00052
*HeCTh* 1000809	*luxazone*	*DrugBank*	*APRD* 00674

### Fragment extraction

Our proposed thesaurus requires a classification scheme in order to be better explored and maintained. Although UMLS-Meta provides a comprehensive taxonomy for the concepts, it cannot be directly applied to our pursued thesaurus due to its complexity and lack of coherence. Indeed, such a taxonomy is the result of merging several thesauri, and therefore it includes many classification criteria. For this reason, we have adopted a *fragment extraction method* [51], which is aimed at retrieving only the taxonomy portion that is involved in the selected vocabulary along with a reduced set of classification concepts. The additional classification concepts can be either manually selected from the integrated thesauri in UMLS-Meta (e.g. Mesh, FMA and SNOMED), or automatically selected from a larger fragment that includes all the ancestors of the vocabulary concepts. In the latter, the selection criterion consists of picking up just those ancestor concepts that cover a minimum number of vocabulary concepts. As shown in the Results section, this strategy is quite effective in extracting reduced and useful fragments.

Regarding other vocabulary sources such as SwissProt and DrugBank, unfortunately they lack a classification scheme as rich as UMLS-Meta. Therefore, it is not possible to apply the *fragment extraction* strategy. To alleviate this problem, we have automatically mapped each of these concepts to the nearest one in UMLS-Meta (refer to Results section). Those concepts without a similar entry in UMLS-Meta are manually classified. This process requires expert intervention in order to both curate mappings and classify non-mapped concepts. In Results section we evaluate the impact of including these sources in the unified lexicon.

**Figure 6:**
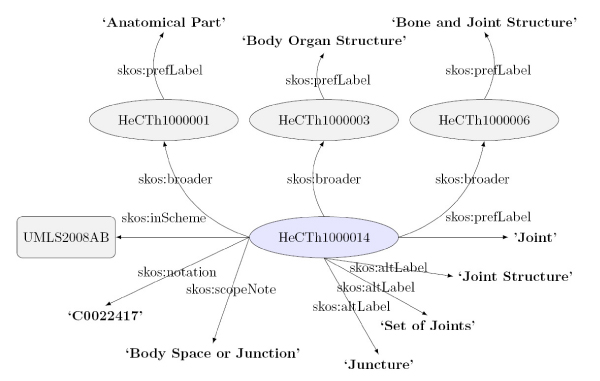
**Example of a SKOS-like HeCTh entry**.

We have used SKOS [52, 53] as a formal language to represent **HeCTh**. This language has a rich support for labeling and reporting term metadata (e.g. Preferred label, Alternate labels, definitions, examples) as well as for defining linguistic relationships (e.g. Has Broader, Has Narrower, Related, Exact Match). Figure 6 shows and example of a SKOS-like **HeCTh** entry which contains an unique entry identifier, the link to the synset (*altLabel*) including the preferred name (*prefLabel*), a basic taxonomy (*broader*) that eases the handling of the thesaurus, a link to a *Semantic Category* (*scopeNote*) and additionally links to external resources, such as UMLS-Meta (*inScheme* and *notation*), have also been included in order to maintain a mapping with the origin thesaurus.

### Thesaurus extension and ontology engineering

The requirements of an ontology may involve concepts with labels that are not present in available lexicons. For example, not all JIA subtypes [21] are properly described in UMLS-Meta. As earlier commented, the shared thesaurus will help ontologies to use a common terminology, but ontologies will also help thesaurus to evolve with concrete necessities. In general, ontologies require a finer granularity than the initially expressed by the thesaurus and will demand the necessity of new concept labels given the specific requirements of the domain. Obviously, a new challenge arises, that is, how to maintain consensual and reference thesaurus up-to-date with respect to the new specific ontologies and their evolutions.

UMLS-Meta adopts the strategy of merging the whole taxonomy and relationships of the evolved information sources, which has several drawbacks as commented previously. However, in our context, the thesaurus taxonomy should be simple enough to allow the incremental evolution of both ontologies and the shared thesaurus. That is, new concepts arisen from ontology construction should be easily updated in the thesaurus and changes in the thesaurus should be quickly notified to ontology designers, as represented in Figure 3.

Currently, **HeCTh** aims at being a reference of three kind of ontologies, each one belonging to a different domain, namely: Juvenile Idiopathic Arthritis (JIA), Tetralogy of Fallot (TOF) and Paediatric Brain Tumors (BT). It is worth mentioning that these ontologies have very different objectives within HeC project. TOF ontologies are mainly aimed at modeling heart anatomy for simulation purposes [54]. BT ontologies are aimed at classifying tumors for prognosis purposes [55, 56]. Finally JIA ontologies are aimed at better classifying patient groups for diagnosis and treatment purposes [21]. Axioms 4 to 6 shows and example of the conceptualization implemented in [50] to classify patients according JIA subtypes.456

Additionally, the thesaurus can be also be applied to annotate patient data forms (i.e. medical protocols) [23]. For this purpose, we regard medical protocols as a set of input controls (input fields in patient data forms), where each control has an associated text label (e.g. *Date of Diagnosis, Bone Erosion Evaluation (BEE)*). Annotations were used to assign a set of terms to each form control. Afterwards, a set of logical representations were associated to each form control in order to use them within a classification purpose ontology (see [23] for a more comprehensive explanation) which aimed to classify controls into categories (e.g. *Medical Procedure*, *Measurement*, etc.).

## Results

In this section we describe the main experimental results achieved in the application of the proposed methodology to the construction of **HeCTh**. As previously mentioned, the vocabulary extraction is mainly guided by medical protocols [23] and related literature [39, 45]. For the latter, we have build three collections of PubMed abstracts, namely: JIA (8,029 abstracts), TOF (7,967 abstracts) and BT (3,666 abstracts). These collections have been semantically indexed with UMLS-Meta 2008AB by using the method proposed in [45]. The number of identified concepts within each collection is indicated in the first column of Table 2.

**Table 2:** Statistics about the concepts obtained for each collection

Collection	Concepts	Frg. Size	Max. Depth	Final Frg.	Max. Depth
JIA	11,577	22,188	42	11,390	12
TOF	9,208	20,684	40	10,669	11
BT	9,732	21,202	45	10,893	11

The second and third columns of the table indicate the features of the fragment extracted from the UMLS-Meta taking into account all the ancestors of the identified concepts in the collection. Notice that the number of concepts is near the double and that the depth of the extracted taxonomy is around 40. As mentioned in the Methods section, we have applied a reduction technique over these fragments in order to keep only relevant ancestors (i.e. those covering at least 15 concepts identified in the collection). Fourth and fifth columns report the features of the resulting fragments. Notice that the reduction is around 43% and that the depth of the taxonomy is also notably reduced.

In order to show the necessity of having just one thesaurus for all the HeC ontologies, we have calculated the overlap between the concepts and documents of the three collections. Notice that the number of shared concepts is relatively high (around 40%, Table 3) whereas the number of shared documents is insignificant. This indicates that even having a set of disjoint collections, the number of shared concepts can be very high. By analysing the shared concepts, we can conclude that they usually involve general biomedicine research methods or very common bioentities (e.g. antibodies, cytokines, antigens, etc.). However, some of the shared concepts correspond to tagger issues (e.g. lead, rise, etc.)

**Table 3:** Overlapping of the three collections.

Pair	Shared Concepts	Shared Docs.
JIA-TOF	4,597 (39.7%)	2
TOF-BT	3,001 (32.6%)	0
JIA-BT	3,354 (28.9%)	1

The second experiment we have carried out was aimed at including other information sources in the **HeCTh**. Specifically, we have selected 18,171 entries from *S* wissProt which are related to human genes or proteins. Then, we have aligned these entries to concepts of UMLS-Meta 2008AB that share some unambiguous lexical token with SwissProt and that have a proper semantic type (e.g. Gene, Protein, etc.).

Specifically, we apply a partial matching approach where the matched part must be unambiguous (i.e. the common part is only present in the matched concepts). As a result, only 310 concepts of SwissProt were not mapped, obtaining thus a coverage of 81%. Comparing our mappings to those provided by UMLS-Meta 2008AB (these mappings stem from HUGO (Human Genome Organization) [57]) the agreement is around 80%. Additionally, our method detects 682 new mappings not regarded by UMLS-Meta. As a result, the alignment between these resources contribute with 15,919 new strings to the UMLS-Meta lexicon, apart from providing the links between these two resources. However, these mappings have little impact in **HeCTh**, as they only affect 3% of its concepts.

Regarding the DrugBank resource, it provides much less entries than UMLS for clinical drugs. However, the entries of UMLS are rather (ontology) instances than concepts. For example for the drug *"methotrexate"*, UMLS-Meta provides 12 terms of the form *"methotrexate 10 mg oral tablet"*, which indicates not only the drug but also the dosage and administration route. In this case, DrugBank seems a better choice to populate the reference thesaurus, keeping UMLS-Meta concepts as links of the resulting entries. For this purpose, similarly to the SwissProt case, an alignment between both sources, UMLS-Meta and DrugBank, has been performed. In this case, resulting mappings are mainly one-to-many due to that the involved UMLS concepts usually represent different variations of the same drug. This alignment is additionally used to organize DrugBank concepts into the UMLS-Meta taxonomy, enriching in this way their organization.

## Conclusion

In this paper we have addressed a still opened issue: the necessity of use and maintenance of a thesaurus for ontology engineering, specially for the Life Sciences. We have also emphasized the main limitations and problems of current resources, which should be better coordinated, integrated and reused.

Our approach for building such a reference thesaurus consists of filtering and re-organizing existing resources and thesauri in order to fit them into the requirements of ontology engineering tools (e.g. text mining, label search, etc...).

We have applied our approach to a real scenario, the Health-e-Child project, and we have evaluated the impact of filtering and re-organizing several knowledge resources. As a result, we have created a thesaurus, named **HeCTh** [50], which partially covers the lexical requirements of the domain ontologies which are being developed in the HeC project. This thesaurus is much simpler in structure and less ambiguous than UMLS-Meta, but richer (in structure) than other resources like SwissProt and DrugBank.

We have filtered terms from existing resources thus not all the domain terms are covered by this approach. An extension of this work will include the extraction of new terms not covered by our thesaurus. The extension of the term coverage will intend to fully cover the domain. These new terms might not have a mapping to existing resources and curation of the extracted terms might be required, even though some automatic approaches have been proposed [58].

Furthermore, the generated thesaurus will be integrated with existing ontologies relevant for HeC which are currently under development. This integration will provide an example of concurrent evolution of these ontologies and the thesaurus. For this purpose, we plan to implement a plug-in to integrate the Protégé ontology lifecycle with **HeCTh** in order to provide an unique and filtered access to the terminological resources so that ontology engineers could easily select the desired term without being overwhelm with several candidates.

Further refinement of these resources will show the benefits and problems driven by the integration presented in this paper. Once the integration in the HeC has been completed we intend to study the feasibility of expanding the coverage of this thesaurus to the biomedical domain.

## List of abbreviations

ACR: (American College of Rheumatology)
BT: (Brain Tumors)
EULAR: (European League Against Rheumatism)
FMA: (Foundational Model of Anatomy)
HeC: (Health-e-Child)
HeCTh: (Health-e-Child Thesaurus)
HUGO: (Human Genome Organization)
ILAR: (International League of Associations for Rheumatology)
JCA: (Juvenile Chronic Arthritis)
JIA: (Juvenile Idiopathic Arthritis)
JRA: (Juvenile Rheumatoid Arthritis)
OBO: (Open Biomedical Ontologies)
OWL: (Ontology Web Language)
SKOS: (Simple Knowledge Organization System)
TA: (Terminologia Anatomica)
TOF: (Tetralogy of Fallot) and UMLS: (Unified Medical Language System).
